# Seasonal Variation in Vitamin D Status Does Not Interfere with Improvements in Aerobic and Muscular Endurance in Conscripts during Basic Military Training

**DOI:** 10.3390/nu16091306

**Published:** 2024-04-26

**Authors:** Saima Timpmann, Leho Rips, Indrek Olveti, Martin Mooses, Hanno Mölder, Ahti Varblane, Hele-Reet Lille, Helena Gapeyeva, Vahur Ööpik

**Affiliations:** 1 Institute of Sport Sciences and Physiotherapy, University of Tartu, 18 Ülikooli St., 50090 Tartu, Estonia; saima.timpmann@ut.ee (S.T.); martin.mooses@ut.ee (M.M.); 2 Sports Medicine and Rehabilitation Clinic, Tartu University Hospital, 1a L. Puusepa St., 50406 Tartu, Estonia; leho.rips@kliinikum.ee; 3 Department of Sports Medicine and Rehabilitation, Institute of Clinical Medicine, Faculty of Medicine, University of Tartu, 18 Ülikooli St., 50090 Tartu, Estonia; 4 Centre of Military Disaster Medicine, Estonian National Defense College, 12 Riia St., 51010 Tartu, Estonia; hele-reet.lille@mil.ee; 5 2nd Infantry Brigade, Estonian Defense Forces, Sirgu Village, Luunja Parish, 62216 Tartu, Estonia; indrek.olveti@mil.ee; 6 Medical Centre of the 2nd Infantry Brigade CSS Battalion, Estonian Defense Forces, 3a Kose Road, 65603 Võru, Estonia; hanno.molder@mil.ee; 7 Joint Headquarters of the Estonian Defense Forces, 58 Juhkentali St., 15007 Tallinn, Estonia; ahti.varblane@mil.ee; 8 Clinic of Medical Rehabilitation, II Rehabilitation Department, East Tallinn Central Hospital, 104 Pärnu St., 11312 Tallinn, Estonia; helena.gapeyeva@itk.ee

**Keywords:** young healthy men, vitamin D status, iron status, ferritin, hemoglobin, 3200 m run, sit-ups, push-ups, testosterone, cortisol

## Abstract

Considering a lack of respective data, the primary objective of this study was to assess whether seasonal variation in vitamin D status (D-status) affects the extent of improvement in physical performance (PP) in conscripts during basic military training (BMT). D-status, PP and several blood parameters were measured repeatedly in conscripts whose 10-week BMT started in July (cohort S-C; *n* = 96) or in October (cohort A-C; *n* = 107). D-status during BMT was higher in S-C compared to A-C (overall serum 25(OH)D 61.4 ± 16.1 and 48.5 ± 20.7 nmol/L, respectively; *p* < 0.0001). Significant (*p* < 0.05) improvements in both aerobic and muscular endurance occurred in both cohorts during BMT. Pooled data of the two cohorts revealed a highly reliable (*p* = 0.000) but weak (R^2^ = 0.038–0.162) positive association between D-status and PP measures both at the beginning and end of BMT. However, further analysis showed that such a relationship occurred only in conscripts with insufficient or deficient D-status, but not in their vitamin D-sufficient companions. Significant (*p* < 0.05) increases in serum testosterone-to-cortisol ratio and decreases in ferritin levels occurred during BMT. In conclusion, a positive association exists between D-status and PP measures, but seasonal variation in D-status does not influence the extent of improvement in PP in conscripts during BMT.

## 1. Introduction

Vitamin D is considered a unique nutrient for humans because it is both absorbed from food and synthesized endogenously [1,2,3]. Moreover, endogenous synthesis in the skin under the influence of solar ultraviolet B radiation is usually the main source of vitamin D for the body [1,4,5], and in fact the physiological need for vitamin D can even be completely met endogenously [6]. However, the endogenous synthesis of vitamin D takes place efficiently only when the exposure to sun is sufficient and the angle of sunlight hitting the skin is greater than 45 degrees [7,8]. Therefore, geographic latitude and the season are important factors that significantly influence the stimulatory effect of ultraviolet B radiation on vitamin D synthesis in human skin [1,4,9]. Since the sun’s elevation angle is too low at north and south latitudes above approximately 35 degrees during the winter months, very little or no vitamin D synthesis occurs in the skin at this time of a year [3,9]. The influence of geographic latitude on endogenous vitamin D production together with the fact that only a few foods naturally contain considerable amounts of vitamin D [9,10,11] may explain why the prevalence of vitamin D deficiency in European countries is twice as high in November–March as in April–October [12]. 

Vitamin D status is evaluated based on serum 25-hydroxyvitamin D (25(OH)D, calcidiol) levels [13,14]. However, the biologically active form is 1,25-dihydroxyvitamin D (1,25(OH)_2_D, calcitriol), which functions in human body via vitamin D receptors (VDRs) like the steroid hormone [11,15,16]. The presence of VDRs has been detected in nearly all cells and tissues in the human body [17], with the highest content occurring in intestine, kidney, parathyroid gland, and bone [18]. Calcitriol, acting via VDRs, exerts both genomic and non-genomic effects [19,20,21]. Costa et al. [22], and Bischoff et al. [23] were the first who demonstrated the expression of VDRs in human skeletal muscle. These findings have been disputed [24,25], but nowadays the presence of VDRs in muscle is considered proven [8,26] and it is well recognized that calcitriol may modify the transcription of a range of muscle proteins (with a slow, genomic effect) and regulate the function of membrane calcium channels (with a rapid, non-genomic effect) [27,28,29].

Cannell et al. [30] were among the first to pay attention to the seasonality of physical performance and, based on the analysis of the world literature, to conclude that vitamin D can significantly affect physical and athletic performance. They suggested that peak athletic performance may occur when serum 25(OH)D levels approach at least 50 ng/mL (125 nmol/L). Since then, the potential effect of vitamin D on athletic performance has been actively investigated but, considering the conclusions of narrative reviews [2,31,32] and the recent meta-analyses [33,34,35], it appears that the relationship between vitamin D and physical performance in athletes has remained unclear. 

Compared to athletes, there are fewer scientific data on the relationship between vitamin D status and physical performance in military personnel. Nevertheless, Carswell et al. [36] studied young healthy military recruits and found that serum 25(OH)D levels did not correlate with muscular strength or power, but were positively associated with endurance running performance. However, recently, Heileson et al. [37] found that serum 25(OH)D levels positively correlated with muscular strength, both muscular and aerobic endurance, and the total Army Fitness Test score in young Reserve Officers Training Corps cadets. Similarly, Laaksi et al. [38] reported a positive relationship between serum 25(OH)D levels and both muscular and aerobic endurance in young Finnish men participating in compulsory military service. They also observed higher testosterone concentrations in men with serum 25(OH)D levels higher than 75 nmol/L compared to their counterparts with lower vitamin D status, and higher serum 25(OH)D levels in participants studied in July compared to those who were studied in January. Ööpik et al. [39] observed an extensive increase in the prevalence of vitamin D deficiency in male Estonian conscripts from October (42.6%) to December (80.8%) but presented no data on the potential associations between vitamin D status and physical performance measures. The findings of a decrease in serum 25(OH)D levels in US female soldiers during military training in late summer and early autumn suggest that not only season, but military training itself, may impair vitamin D status in military personnel [40].

Thus, there is some evidence for a positive association between vitamin D status and physical performance in military personnel, as well as on the effect of season and potentially that of military training on vitamin D status. However, there appears to be a lack of data on the possible impact of seasonal variability in vitamin D status on the extent of improvement in performance indicators during military training. Therefore, the primary objective of this study was to assess whether seasonal variation in vitamin D status affects the extent of improvement in physical performance in conscripts during basic military training (BMT). The secondary objective was to evaluate associations between vitamin D status and indicators of muscular and aerobic endurance, and the possible effect of BMT on these associations.

## 2. Materials and Methods

### 2.1. Participants and Study Design

The study was carried out on two cohorts of young Estonian men during the first 10 weeks of their compulsory military service in the Kuperjanov Single Infantry Battalion. All conscripts entering military service were invited to participate in the study. They were informed about the purpose of the study, the related procedures, the time the procedures would take, and also the voluntary nature of their participation. Conscription of one cohort began in early October 2014, with 107 of its 407 members agreeing to participate in the study (hereinafter referred to as the “autumn cohort”; A-C). The military service of the second cohort, of which 96 of the 410 men agreed to participate in the study, began in July 2015 (hereinafter the “summer cohort”; S-C). At the beginning of their participation in the study the age, height, body mass and body mass index of the A-C and S-C were (mean ± SD) 20.9 ± 1.7 and 21.0 ± 1.6 years, 182.1 ± 6.4 and 181.7 ± 6.3 cm, 80.5 ± 11.3 and 80.1 ± 11.2 kg, and 24.3 ± 3.1 and 24.2 ± 2.9 kg/m^2^, respectively.

All conscripts, including the study participants, had to pass a 10-week BMT according to the standard program established by the Command of the Estonian Defense Forces. Food and water intake were in accordance with the standard army meal, the conscripts slept in dormitory-type rooms and the average sleeping time was 8 h. During BMT, conscripts were daily involved in physically demanding activities, as described by Ööpik et al. [39]. There were no essential differences between A-C and S-C in terms of the physical demands of BMT, food supply, or the ratio of daily physical activity and rest time.

During BMT, several study participants experienced temporary illness, mental health problems, back pain, limb injuries, etc., and it was not possible to collect all the planned data on them. Therefore, when presenting the results, the specific number of conscripts is also indicated for all data.

The study protocol was in accordance with the principles of the Declaration of Helsinki, and it was approved by the Research Ethics Committee of the University of Tartu (protocols no. 239/T-15, 25 August 2014 and no. 249/M-26, 15 June 2015, respectively). 

### 2.2. Blood Sampling and Analyses

The participants were repeatedly subjected to blood tests and twice, during the first and eighth weeks of BMT, data on their physical performance were collected.

In A-C, each participant donated a total of five venous blood samples: one, two, six, ten and twenty-five weeks after the start of the BMT. Participants in S-C gave six blood sample altogether: one, three, seven, eleven, twenty-four and thirty-nine weeks after the start of the BMT. Thus, in both A-C and S-C, four blood samples were collected during or shortly after the 10-week BMT. The inconsistent timing of blood sampling in the two cohorts was not intentional, but was due to logistical reasons. Nevertheless, all blood samples were taken after a two-day rest, on the Monday morning before breakfast, with participants weighed just before this procedure.

Blood was collected into 5-mL Vacutainer serum tubes as well as into 3-mL Vacutainer tubes containing EDTA (Becton, Dickinson & Co., Franklin Lakes, NJ, USA). The blood from the EDTA tube was used for hemogram measurement using the analyzer Sysmex XE-2100D (Sysmex Corporation, Kobe, Japan). Blood collected into serum tubes was first allowed to clot, after which the serum was separated by centrifugation. After centrifugation, the tubes were maintained at 4 °C and transported to the United Laboratories of the Tartu University Hospital for analysis. 

In serum, the concentrations of testosterone, cortisol, ferritin and 25(OH)D were determined. The chemiluminescent immunoassay (CLIA) method was used for measurement of serum ferritin and testosterone (Siemens Centaur XP, Siemens Healthcare GmbH, Erlangen, Germany), cortisol (Immulite 2000 XP, Siemens Healthcare GmbH, Erlangen, Germany) and 25(OH)D (Liaison XL, DiaSorin S.p.A, Saluggia, VC, Italy) concentrations.

Based on serum 25(OH)D concentration, the participants were classified as being vitamin D sufficient (≥75 nmol/L), insufficient (<75 ≥50 nmol/L), or deficient (<50 nmol/L). Similar criteria for assessing the vitamin D status of military personnel have been previously applied by Funderburk et al. [41]. The cut-off value used for identifying vitamin D deficiency was that recommended by the Endocrine Society [13]. 

The criteria applied for identifying iron deficiency and iron deficiency anemia were serum ferritin level ≤ 35 μg/L and blood hemoglobin concentration < 120 g/L, respectively [42]. Ferritin concentration ≤ 35 μg/L with concomitant hemoglobin levels > 120 g/L were classified as iron deficiency without anemia [42]. 

### 2.3. Assessment of Physical Performance

According to the standard BMT program established by the Command of the Estonian Defense Forces, all participants completed a physical fitness test (PFT) two times: during the first (Test 1) and eighth (Test 2) weeks of the BMT. The PFT used is known as the US Army Physical Fitness Test and it is designed to measure upper-body and core muscular endurance, aerobic endurance, and cardiorespiratory health [37,43]. Thus, participants had to perform as many push-ups and sit-ups as they could in two separated 2 min periods and complete a timed 3200 m run. In both A-C and S-C, both testing sessions took place on the same day and on the same running track under similar conditions. The results were recorded by the experienced staff of the Infantry Battalion.

### 2.4. Statistical Analysis

For statistical processing of the collected data, the software program Statistica 13.3 (TIBCO Software Inc., Palo Alto, CA, USA) was used. Data are presented as mean ± SD. The normal distribution of data was checked by the Kolmogorov–Smirnov test. A two-way repeated analysis of variance ANOVA with a factor between cohorts (A-C vs. S-C) and within a time factor was used to evaluate the differences within and between the cohorts. If a significant main effect or interaction occurred, Tukey’s honestly significant difference post hoc analysis was used to locate differences between the means. Partial η-squared (η_p_^2^) is reported as a measure of effect size. A small effect was reported for η_p_^2^ > 0.01, a medium effect for η_p_^2^ > 0.06, and a large effect for η_p_^2^ ≥ 0.14. The mean values of different parameters registered at a single time point were compared using Student’s *t* test for independent variables. Significance was set at the *p* < 0.05 level. A Pearson product moment coefficient of correlation (*r*) was applied to determine the relationship between variables.

## 3. Results

**Figure 1:**
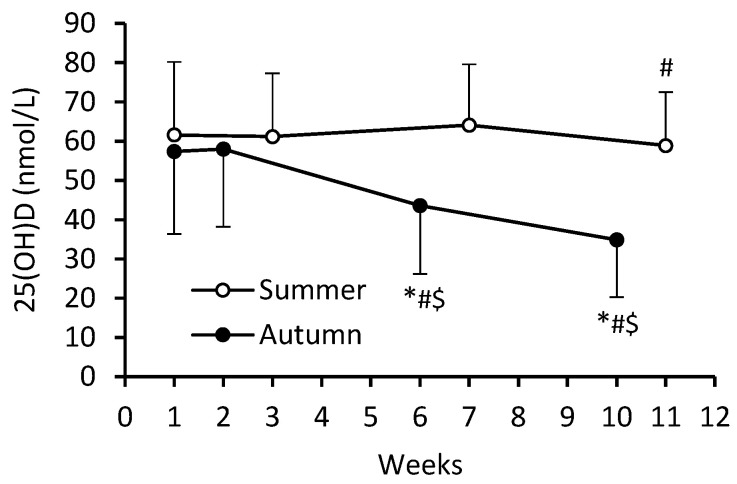
Serum 25(OH)D concentrations during BMT in summer (S-C) and autumn (A-C) cohorts. Data are presented as mean ± SD; *n* = 84 in S-C and *n* = 99 in A-C. The numbering of weeks begins from the first week of BMT, which for S-C was at the beginning of July and for A-C at the beginning of October. The last time point (week 11 for S-C and week 10 for A-C) was in mid-September and at the beginning of December, respectively. Significantly different (*p* < 0.05): * from week 1; ^#^ from previous time point; ^$^ from S-C.

There were significant main effects for cohort (*F* = 28.62; η_p_^2^ = 0.136) and time (*F* = 143.16; η_p_^2^ = 0.442), and a significant cohort–time interaction (*F* = 115.97; η_p_^2^ = 0.390) for serum 25(OH)D concentrations (in all cases, *p* < 0.0001). Overall, serum 25(OH)D levels were higher in S-C compared to A-C during BMT (61.4 ± 16.1 vs. 48.5 ± 20.7 nmol/L; *p* < 0.0001). At week 1, serum 25(OH)D concentrations were similar for the two cohorts, but from mid-BMT (week 6 for A-C and week 7 for S-C) to the end of BMT, serum 25(OH)D levels were significantly lower in A-C. Throughout BMT, serum 25(OH)D levels decreased significantly (39.2%; *p* < 0.0001) in A-C, but remained virtually unchanged in S-C. The largest between-cohort difference occurred at the end of BMT (58.9 ± 13.6 vs. 34.9 ± 14.6 nmol/L, in S-C and A-C, respectively; *p* < 0.0001) (Figure 1).

The proportion of vitamin D-insufficient and -deficient conscripts was high in both S-C and A-C: 79.8% and 79.4% at the beginning of BMT, and 88.2% and 98% at the end of the BMT, respectively. The last blood samples for the 25(OH)D analysis were taken from conscripts in both cohorts towards the end of March. By that time, regardless of the cohort, the vitamin D status of all conscripts, without exception, had fallen to a level of insufficiency or deficiency. 

**Figure 2:**
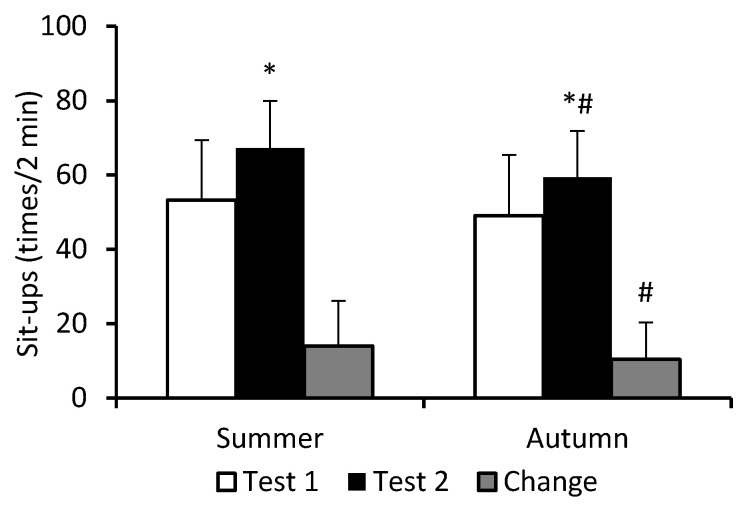
Performance on sit-up exercise. Data are presented as mean ± SD; *n* = 82 in summer cohort and *n* = 95 in autumn cohort. Test 1—first week of BMT, Test 2—eighth week of BMT. Change refers to the extent of improvement in performance in Test 2 compared to Test 1. Significantly different (*p* < 0.05): * from Test 1; ^#^ from summer.

Significant main effects for cohort (*F* = 8.85; *p* = 0.003; η_p_^2^ = 0.048) and test (*F* = 217.83; *p* < 0.0001; η_p_^2^ = 0.554), and a significant cohort–test interaction (*F* = 4.68; *p* = 0.032; η_p_^2^ = 0.026) occurred in the performance of the sit-up exercise. During BMT, performance of this exercise improved significantly in both cohorts. However, the improvement was greater in S-C (26%) than in A-C (21%) (Figure 2). 

**Figure 3:**
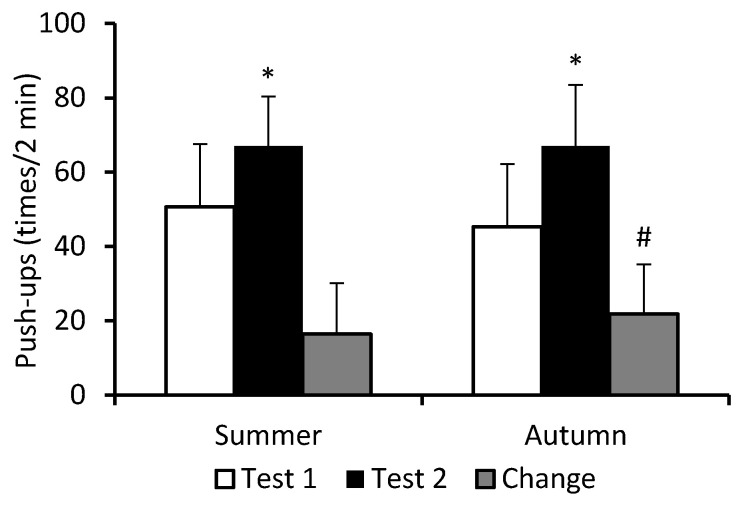
Performance on push-up exercise. Data are presented as mean ± SD; *n* = 82 in summer cohort and *n* = 95 in autumn cohort. Test 1—first week of BMT, Test 2—eighth week of BMT. Change refers to the extent of improvement in performance in Test 2 compared to Test 1. Significantly different (*p* < 0.05): * from Test 1; ^#^ from summer.

There was a significant main effect of test (*F* = 351.25; *p* < 0.0001; η_p_^2^ = 0.669), and a significant cohort–test interaction (*F* = 6.94; *p* = 0.009; η_p_^2^ = 0.038), but no effect of cohort (*F* = 1.35; *p* = 0.247; η_p_^2^ = 0.008) for performance of the push-up exercise. During BMT, performance on this exercise also improved significantly in both cohorts, but unlike the sit-up task, improvement was greater in A-C (48%) than in S-C (32%) (Figure 3).

**Figure 4:**
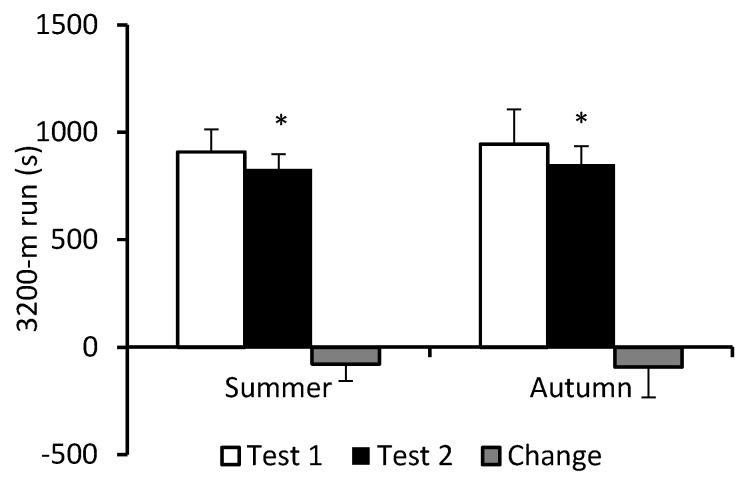
Performance on 3200 m timed run. Data are presented as mean ± SD; *n* = 82 in summer cohort and *n* = 92 in autumn cohort. Test 1—first week of BMT, Test 2—eighth week of BMT. Change refers to the extent of improvement in performance in Test 2 compared to Test 1. Significantly different (*p* < 0.05): * from Test 1.

Only a main effect of test (*F* = 96.95; *p* < 0.0001; η_p_^2^ = 0.360) occurred for performance on timed 3200-m run. During BMT, performance on this exercise improved significantly and to a similar extent in S-C (9%) and A-C (10%) (Figure 4).

**Figure 5:**
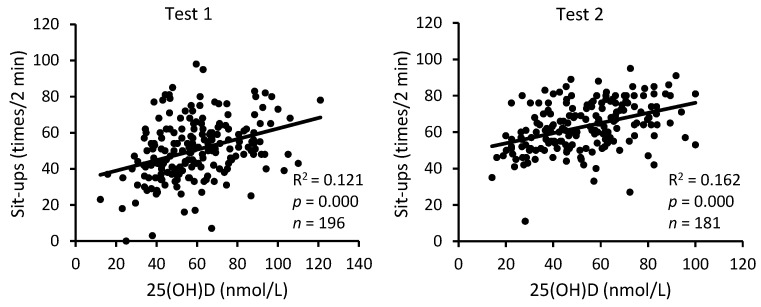
Relationship between serum 25(OH)D levels and performance: sit-up exercise. Test 1—first week of BMT, Test 2—eighth week of BMT.

**Figure 6:**
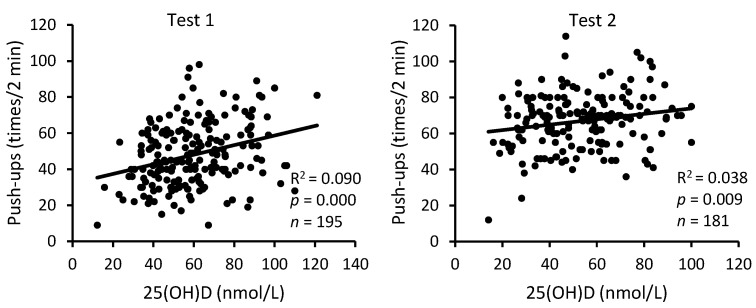
Relationship between serum 25(OH)D levels and performance: push-up exercise. Test 1—first week of BMT, Test 2—eighth week of BMT.

**Figure 7:**
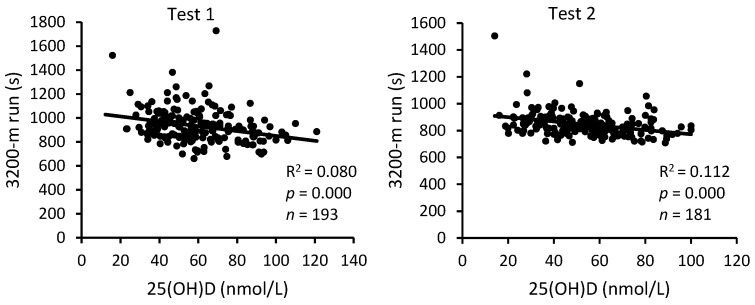
Relationship between serum 25(OH)D levels and performance: 3200 m run. Test 1—first week of BMT, Test 2—eighth week of BMT.

The analysis of pooled data of S-C and A-C revealed a statistically highly reliable relationship between serum 25(OH)D levels and the three physical performance indicators, both at the beginning and at the end of BMT (Figure 5, Figure 6 and Figure 7). The squared values of the correlation coefficients suggest that at the beginning of BMT, serum 25(OH)D levels may explain approximately 12%, 9%, and 8% of the inter-individual variability in performance on sit-up and push-up exercises and the 3200 m run, respectively. At the end of the BMT, the corresponding figures were 16%, 4% and 11%.

For a more detailed analysis, the serum 25(OH)D data were stratified according to the vitamin D status of the participants. Thus, it turned out that a statistically reliable relationship between serum 25(OH)D levels and the three performance indicators occurred only in conscripts with vitamin D insufficiency or deficiency, but not in their companions with sufficient-vitamin D status (Table 1). 

**Figure 8:**
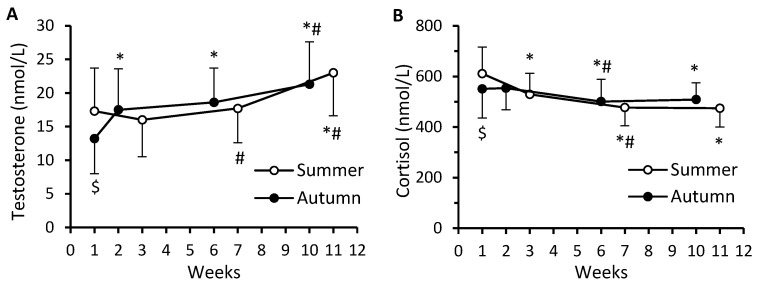
Serum concentrations of testosterone (**A**) and cortisol (**B**). Data are presented as mean ± SD; *n* = 84 in summer cohort and *n* = 99 in autumn cohort. The numbering of weeks begins from the first week of BMT, which in S-C was at the beginning of July and in A-C at the beginning of October. The last time point (week 11 for S-C and week 10 for A-C) was in mid-September and at the beginning of December, respectively. Significantly different (*p* < 0.05): * from week 1; ^#^ from previous time point; ^$^ from summer.

**Figure 9:**
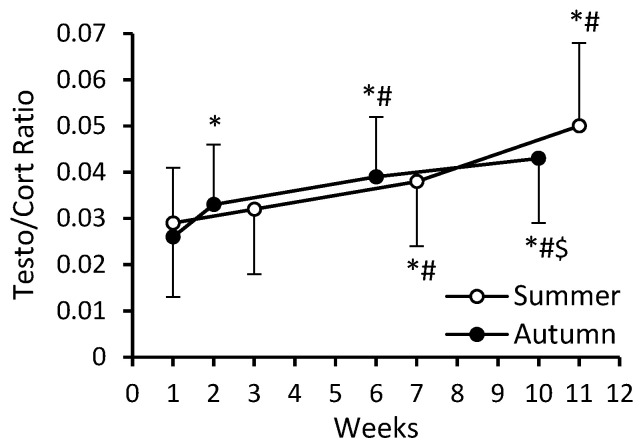
The testosterone-to-cortisol ratio. Data are presented as mean ± SD; *n* = 84 in summer cohort and *n* = 99 in autumn cohort. The numbering of weeks begins from the first week of BMT, which in S-C was at the beginning of July and for A-C at the beginning of October. The last time point (week 11 for S-C and week 10 for A-C) was in mid-September and at the beginning of December, respectively. Significantly different (*p* < 0.05): * from week 1; ^#^ from previous time point; ^$^ from summer.

A significant main effect of time occurred for both serum testosterone (*F* = 172.49; *p* < 0.0001; η_p_^2^ = 0.488) and cortisol levels (*F* = 82.42; *p* < 0.0001; η_p_^2^ = 0.313). There was also a significant cohort–time interaction (*F* = 34.04; η_p_^2^ = 0.158 and *F* = 20.83; η_p_^2^ = 0.103; *p* < 0.0001) for testosterone and cortisol, respectively. At the beginning of BMT, the levels of both testosterone and cortisol were higher in S-C than in A-C (Figure 8A,B). The changes in testosterone and cortisol levels were similar in S-C and A-C and resulted in significant increases in testosterone (32.9% and 61.4%, respectively) and decreases in cortisol (22.3% and 7.6%, respectively) by the end, compared to the beginning of BMT (Figure 8A,B). A significant main effect of time (*F* = 177.71; *p* < 0.0001; η_p_^2^ = 0.495), and a significant cohort–time interaction (*F* = 10.12; *p* < 0.0001; η_p_^2^ = 0.053) occurred for the testosterone-to-cortisol ratio (TCR) (Figure 9). During BMT, the TCR increased in both S-C and A-C, by 72.4% and 65.4% respectively.

The correlation analysis, which included pooled data (*n* = 193) from both S-C and A-C at the beginning (*r* = 0.005; *p* = 0.947) and at the end (*r* = –0.072; *p* = 0.323) of BMT, showed no association between serum 25(OH)D and testosterone levels.

**Figure 10:**
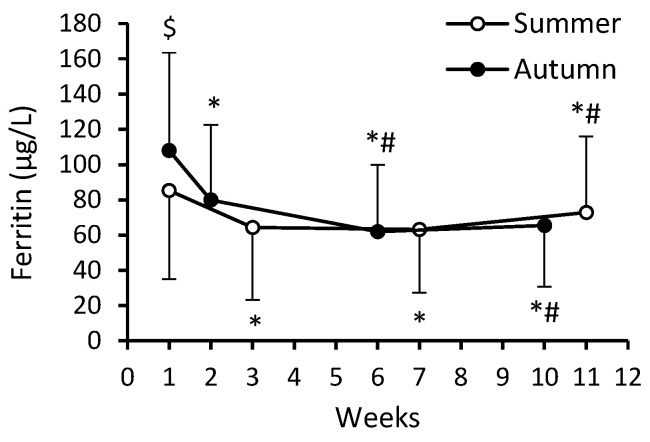
Serum ferritin concentration. Data are presented as mean ± SD; *n* = 84 in summer cohort and *n* = 99 in autumn cohort. The numbering of weeks begins from the first week of BMT, which in S-C was at the beginning of July and in A-C at the beginning of October. The last time point (week 11 for S-C and week 10 for A-C) was in mid-September and at the beginning of December, respectively. Significantly different (*p* < 0.05): * from week 1; ^#^ from previous time point; ^$^ from summer.

A significant main effect of time (*F* = 131.95; *p* < 0.0001; η_p_^2^ = 0.422) and cohort–time interaction (*F* = 29.44; *p* < 0.0001; η_p_^2^ = 0.140) occurred for serum ferritin levels. At the beginning of BMT, serum ferritin concentration was lower in S-C compared to A-C (85.3 ± 50.3 μg/L vs. 108.0 ± 55.5 μg/L, respectively), but no significant between-cohort differences occurred at any time points (Figure 10). During the first weeks of BMT, the ferritin level decreased in both cohorts and remained lower compared to week 1 until the end of the BMT by 14.6% (S-C) and 39.4% (A-C). In conscripts for whom the results of the 3200 m run and for the ferritin data from both the beginning and the end of BMT were available (*n* = 172), more extensive decreases in ferritin levels were correlated with greater increases in aerobic performance during BMT (*r* = 0.217; *p* = 0.004). During BMT, the number of participants with serum ferritin levels ≤ 35 μg/L was eight (9.5%) in S-C and eight (8.1%) in A-C.

No significant between-cohort differences occurred in hemoglobin concentration (*F* = 0.781; *p* = 0.378; η_p_^2^ = 0.004) or hematocrit (*F* = 1.322; *p* = 0.252; η_p_^2^ = 0.007) during the BMT. Hemoglobin and hematocrit values in the two cohorts did not differ at any time point (Table 2). In S-C, the hemoglobin levels remained stable throughout the BMT, while in A-C an increase of 3.7% compared to the beginning of the BMT occurred. All eight conscripts in S-C, whose serum ferritin levels were consistently ≤ 35 μg/L, had a hemoglobin concentration > 120 g/L. Of the eight conscripts in A-C, with ferritin level ≤ 35 μg/L, only one had a hemoglobin concentration < 120 g/L. During BMT, hematocrit increased by 2.3% (S-C) and 2.2% (A-C).

## 4. Discussion

The primary objective of this study was to assess whether seasonal variation in vitamin D status affects the extent of improvement in physical performance in conscripts during BMT. The secondary objective was to evaluate associations between vitamin D status and indicators of muscular and aerobic endurance, and the possible effect of BMT on these associations.

The four main findings of the present study are the following: (1) overall higher serum 25(OH)D levels in S-C compared to A-C during BMT; (2) an absence of a clear effect of seasonal variability in vitamin D status on the extent of improvement in physical performance indicators during BMT; (3) at the level of pooled data of the two cohorts, highly reliable associations between serum 25(OH)D levels and physical performance indicators both at the beginning and at the end of BMT; and (4) an absence of association between serum 25(OH)D levels and physical performance indicators in the subgroup of participants with serum 25(OH)D levels ≥ 75 nmol/L.

Higher overall serum 25(OH)D levels observed in S-C compared to A-C was an anticipated outcome because Estonia is located at high latitudes of 57°37′–59°49′ N and seasonal variation in vitamin D status has been well elucidated in the general population of this country [44]. Similarly, Laaksi et al. [38] reported higher serum 25(OH)D levels in young male Finnish military personnel studied in summer compared to those tested in winter. Nevertheless, some data suggest that high loads of military or athletic training per se may exert a negative impact on vitamin D status, independently of the season. For example, Andersen et al. [40] observed a significant 13% decline in serum 25(OH)D levels in female US army personnel during summertime 8-week basic combat training. On the other hand, Koundourakis et al. [45] found a significant 37% increase in serum 25(OH)D concentrations in professional soccer players during a 6-week off-season period compared to the levels observed at the end of the preceding competition period, and concluded that reductions in exercise training stress may have beneficial effects on vitamin D status. However, it is unlikely that the 27% higher overall serum 25(OH)D level in our S-C group compared to A-C resulted from different training loads in the two cohorts, as the BMT program is basically the same in Estonian conscripts, regardless of the time of year. Both the food intake and the rest-and-sleep regime were also consistent with general army standards and did not differ between our two conscript cohorts during BMT.

Sit-up and push-up exercise tests measure muscular endurance in different muscle groups [37]. During BMT, performance in both sit-up and push-up tests improved in both cohorts, while in the sit-up test the extent of improvement was greater in the S-C and in the push-up exercise the A-C exhibited greater positive change. If vitamin D status had been an important factor influencing the improvement of muscular endurance, then the results of the push-up test should also have improved more in the S-C than in A-C. Thus, although vitamin D status was higher in the S-C due to the more favorable season, this did not influence the extent of improvement in muscular endurance. The reasons why muscular endurance improved to a different extent in the sit-up and push-up tests in the two cohorts are not clear. Because these exercises involve different muscle groups, it can be assumed that they were loaded to somewhat different degrees in the two cohorts of conscripts during BMT. Some variability in the specifics of training loads cannot be ruled out, although basically the BMT program is the same for all conscripts.

The timed 3200 m run is considered a reliable test for assessing aerobic endurance and cardiopulmonary health in military personnel [37]. Running performance improved to a similar extent in our two cohorts of conscripts, suggesting that, as in case of muscular endurance, vitamin D status did not influence progression in aerobic endurance and cardiopulmonary health during BMT. Taken together, our data show that the improvements in muscular and aerobic performance during BMT were similar in the two cohorts of conscripts, despite significantly lower serum 25(OH)D levels observed in A-C compared to S-C due to season.

At the level of pooled data of S-C and A-C, a statistically highly reliable positive relationship occurred between serum 25(OH)D levels and the three physical performance indicators, both at the beginning and at the end of the BMT. This finding is in direct contrast to data from Barringer et al. [46], who employed the exact same battery of tests in a study of active-duty soldiers but found no association between vitamin D status and performance in any of the three exercises. The reasons for the discrepancy between our data and that of Barringer et al. [46] remain obscure. However, like us, other research groups [36,37,38] observed a positive relationship between vitamin D status and aerobic endurance in military personnel. Regarding the positive association between vitamin D status and muscular endurance, our data are consistent with those of Heileson et al. [37] and Laaksi et al. [38]. Nevertheless, there is some novelty in our data because we evaluated the relationship between vitamin D status and physical performance indicators both at the beginning and at the end of the BMT, whereas the other research groups [36,37,38,46] did this only once. Since for most Estonian conscripts, BMT can be considered as the first experience in demanding systematic physical training [39], our data suggest that the relationship between vitamin D status and physical performance is stable and that relatively high training loads do not disrupt it.

On the other hand, stratification of the pooled data by serum 25(OH)D levels showed that the association between vitamin D status and physical performance measures existed only when the serum 25(OH)D concentrations were below 75 nmol/L. According to the American Endocrine Society, a serum 25(OH)D level of 75 nmol/L differentiates between vitamin D sufficiency and insufficiency [13]. However, the Institute of Medicine [14] and the National Osteoporosis Society [47] consider a serum 25(OH)D level of 50 nmol/L or higher to be sufficient. When we set the serum 25(OH)D cut-off between vitamin D sufficiency and insufficiency at 50 nmol/L, the pattern of the relationship between vitamin D status and physical performance measures was no longer as clear as when using a cut-off value of 75 nmol/L. Thus, given the divergent views on the cut-off level of serum 25(OH)D defining vitamin D sufficiency, our data are in line with the position that it is 75 nmol/L, rather than 50 nmol/L.

Hormones have an important role in eliciting physiological adaptations to exercise training, and acute as well as chronic changes in serum hormone levels enable the evaluation of the level of training stress. Therefore, the monitoring of various hormonal markers can provide valuable information about the efficacy of the training process and the development of the trained state [48,49]. In a military environment, monitoring of serum testosterone and cortisol levels or TCR has been used for evaluation of training stress and for detecting too-heavy training loads which may lead to maladaptation [50,51,52,53]. In our conscripts, in both S-C and A-C, a significant increase in TCR over time suggests that they tolerated well the BMT-induced physical and psychological stress. This is also confirmed by the fact that all three physical performance measures improved significantly during BMT.

Previous studies [54,55] and a recent large-scale meta-analysis [56] have revealed a positive association between vitamin D status and serum testosterone levels. In addition to a positive association, Wehr et al. [57] reported similar seasonal variation in serum 25(OH)D and testosterone concentrations. When analyzing pooled S-C and A-C data, we did not observe an association between serum 25(OH)D and testosterone levels. At first glance, this finding contradicts the data of Damas-Fuentes et al. [54], Nimptsch et al. [55], and Wehr et al. [57] and the general conclusion of a large-scale meta-analysis [56]. However, all our participants were healthy young men, whereas Damas-Fuentes et al. [54] reported a significant positive association between serum 25(OH)D and testosterone levels only in men with morbid obesity, and Nimptsch et al. [55] and Wehr et al. [57] observed such an association in middle-aged and elderly men. A meta-analysis by D’Andrea et al. [56] included data from eighteen studies involving 20,576 individuals altogether. However, only in two studies, comprising merely 2.2% of the individual cases analyzed, was the mean age of participants below 30 years, i.e., like our conscripts. More importantly, the authors noted that a significant positive association between 25(OH)D and total testosterone levels occurred in the seven studies carried out with frail participants, whereas studies enrolling non-frail populations revealed no association between 25(OH)D and testosterone [56]. Interestingly, Laaksi et al. [38] recently reported a weak but statistically significant negative association between serum 25(OH)D and testosterone levels in Finnish conscripts of similar age to our participants. The reasons for the discrepancy between our data and those of Laaksi et al. [38] remain unclear, but the possible relationship between vitamin D status and testosterone levels in young healthy men deserves further studies, due to the paucity of available data.

Hemoglobin is an iron-containing protein in erythrocytes that plays a key role in the transport of oxygen from the pulmonary alveoli to the tissues and of carbon dioxide from tissues to the pulmonary alveoli [58]. Ferritin is an iron-storing protein [59], the serum level of which reflects the iron status of the body [60]. Suboptimal blood levels of both hemoglobin [61,62] and ferritin [42] are associated with impaired aerobic endurance. In our conscripts, hemoglobin levels did not change during BMT in the S-C, but there was a small (3.7%) statistically significant increase in the A-C. Contrary to our data, other research groups [63,64,65] observed a decrease in hemoglobin levels in male soldiers during BMT, lasting from 9 weeks to 4 months. The discrepancy between our findings and those of the previous studies may be due to many factors, such as the duration of training, the proportions of training loads with different goals, nutrient intake, and the physical performance of soldiers before the start of military training.

On the other hand, a significant decrease in ferritin levels observed during BMT in both S-C (14.6%) and A-C (39.4%) is in good agreement with previous data [63,64,65,66]. With a background of relatively stable hemoglobin concentrations, a decrease in ferritin levels did not prevent the improvement in aerobic endurance during BMT in our conscripts. On the contrary, larger decreases in ferritin levels were associated with greater improvements in the 3200 m-run time during BMT. The same phenomenon, a positive association between a decrease in ferritin levels and improvement in aerobic endurance during military training, was recently noted by O’Leary et al. [64]. Ferritin is considered a biomarker of iron stored in the bone marrow, liver, and spleen [67,68], while the portion of iron that transports and uses oxygen in the production of energy is known as functional iron [69]. Thus, decreased ferritin levels concomitant with improved aerobic endurance may reflect the mobilization of iron from storage sites into the functional compartment in our conscripts. On the other hand, most of the functional iron is contained in hemoglobin and myoglobin [70], but hemoglobin levels in our conscripts remained unchanged (S-C) or only slightly increased (A-C), and exercise is not known to increase myoglobin levels in human skeletal muscle [71]. However, exercise stimulates the synthesis of iron-containing mitochondrial proteins in muscle, including components of the respiratory chain and enzymes involved in oxidation processes at the substrate level [67,72]. Thus, BMT-induced adaptations at the level of skeletal muscle mitochondria may explain the positive association between decreased serum ferritin and improved aerobic endurance in our conscripts.

One of the strengths of this study is its design, which allowed us to assess the potential impact of seasonal variability in vitamin D status on the effectiveness of BMT in improving physical performance in conscripts. To our knowledge, this is the first study of its kind. In addition, since the daily life of conscripts such as food consumption and the BMT program was uniform, many unmeasured potential confounders were kept under control. Also, the timing of blood sampling on Monday morning, i.e., after 2 days of rest and approximately 12 h of overnight fasting, allowed us to assess the chronic effects of the BMT program on blood parameters without the confounding effects of acute exercise. An important limitation of this study is the absence of data on the leisure time physical activity of conscripts. Additionally, one problem with the current study is that the number of conscripts with adequate vitamin D status was quite small. Therefore, our data on the relationship between serum 25(OH)D levels and indicators of physical performance in this subgroup of conscripts should be interpreted with caution. It is also worth noting that we only studied young men, so our results cannot be generalized to female military personnel. 

## 5. Conclusions

In summary, our results suggest that seasonal variation in vitamin D status does not influence the extent of improvement in aerobic and muscular endurance in young male conscripts during BMT. A highly reliable but weak positive association occurs between serum 25(OH)D levels and physical performance measures both at the beginning and end of BMT in conscripts with insufficient or deficient vitamin D status, but not in their vitamin D-sufficient companions. An increase in the serum TCR and a decrease in ferritin levels in conscripts may indicate the occurrence of anabolic adaptation processes and a decrease in body iron stores during BMT, respectively.

## Figures and Tables

**Table 1:** Relationship between serum 25(OH)D levels and physical performance.

25(OH)D	Sit-Ups		Push-Ups		3200 m Run	
	Test 1	Test 2	Test 1	Test 2	Test 1	Test 2
≥75 nmol/L	r = 0.250	r = 0.065	r = 0.156	r = –0.113	r = –0.236	r = –0.102
	R^2^ = 0.064	R^2^ = 0.004	R^2^ = 0.024	R^2^ = 0.013	R^2^ = 0.056	R^2^ = 0.010
	*p* = 0.108	*p* = 0.750	*p* = 0.323	*p* = 0.582	*p* = 0.133	*p* = 0.621
	*n* = 42	*n* = 26	*n* = 42	*n* = 26	*n* = 42	*n* = 26
<75 nmol/L	r = 0.320	r = 0.384	r = 0.280	r = 0.191	r = –0.188	r = –0.361
	R^2^ = 0.102	R^2^ = 0.147	R^2^ = 0.078	R^2^ = 0.036	R^2^ = 0.035	R^2^ = 0.130
	***p* = 0.000**	***p* = 0.000**	***p* = 0.000**	***p* = 0.017**	***p* = 0.021**	***p* = 0.000**
	*n* = 154	*n* = 155	*n* = 153	*n* = 155	*n* = 151	*n* = 155
≥50 nmol/L	r = 0.243	r = 0.268	r = 0.185	r = 0.121	r = –0.128	r = –0.174
	R^2^ = 0.059	R^2^ = 0.072	R^2^ = 0.034	R^2^ = 0.015	R^2^ = 0.016	R^2^ = 0.030
	***p* = 0.006**	***p* = 0.009**	***p* = 0.038**	*p* = 0.244	*p* = 0.145	*p* = 0.092
	*n* = 128	*n* = 95	*n* = 127	*n* = 95	*n* = 128	*n* = 95
<50 nmol/L	r = 0.428	r = 0.413	r = 0.320	r = 0.272	r = –0.131	r = –0.331
	R^2^ = 0.183	R^2^ = 0.171	R^2^ = 0.102	R^2^ = 0.074	R^2^ = 0.017	R^2^ = 0.110
	***p* = 0.000**	***p* = 0.000**	***p* = 0.008**	***p* = 0.011**	*p* = 0.298	***p* = 0.002**
	*n* = 68	*n* = 86	*n* = 68	*n* = 86	*n* = 65	*n* = 86

Test 1—first week of BMT, Test 2—eighth week of BMT. Serum 25(OH)D levels ≥75 nmol/L are considered sufficient, <75 ≥50 nmol/L insufficient, and <50 nmol/L deficient [13,41]. *p* values indicating statistically reliable relationships between serum 25(OH)D levels and physical performance are presented in bold.

**Table 2:** Hematological parameters.

Variable	Cohort	Weeks			
		Week 1	Week 2 or 3	Week 6 or 7	Week 10 or 11
Hemoglobin (g/L)	Summer	149.2 ± 9.7	150.4 ± 9.2	150.5 ± 10.7	149.9 ± 11.3
	Autumn	147.3 ± 9.9	146.3 ± 9.8	149.2 ± 9.1 ^#^	152.7 ± 8.2 *^#^
Hematocrit (%)	Summer	43.6 ± 2.5	44.5 ± 2.3 *	44.2 ± 2.8	44.6 ± 2.9 *
	Autumn	44.6 ± 2.5	43.6 ± 2.4 *	44.6 ± 2.2 ^#^	45.6 ± 2.1 *^#^

Data are presented as mean ± SD; *n* = 84 in summer cohort and *n* = 99 in autumn cohort. Significantly different (*p* < 0.05): * from week 1; ^#^ from previous time point.

## Data Availability

The data presented in this study are available on request from the corresponding author (vahur.oopik@ut.ee).

## References

[1] Bendik I., Friedel A., Roos F.F., Weber P., Eggersdorfer M. (2014). Vitamin D: A critical and essential micronutrient for human health. Front. Physiol..

[2] Owens D.J., Fraser W.D., Close G.L. (2015). Vitamin D and the athlete: Emerging insights. Eur. J. Sport Sci..

[3] Zittermann A., Trummer C., Theiler-Schwetz V., Lerchbaum E., März W., Pilz S. (2021). Vitamin D and cardiovascular disease: An updated narrative review. Int. J. Mol. Sci..

[4] Janoušek J., Pilařová V., Macáková K., Nomura A., Veiga-Matos J., Silva D.D.D., Remião F., Saso L., Malá-Ládová K., Malý J. (2022). Vitamin D: Sources, physiological role, biokinetics, deficiency, therapeutic use, toxicity, and overview of analytical methods for detection of vitamin D and its metabolites. Crit. Rev. Clin. Lab. Sci..

[5] Owens D.J., Allison R., Close G.L. (2018). Vitamin D and the athlete: Current perspectives and new challenges. Sports Med..

[6] Willis K.S., Peterson N.J., Larsom-Meyer D.E. (2008). Should we be concerned about the vitamin D status of athletes?. Int. J. Sport Nutr. Exerc. Metab..

[7] Bishop E.L., Ismailova A., Dimeloe S., Hewison M., White J.H. (2021). Vitamin D and immune regulation: Antibacterial, antiviral, anti-inflammatory. JBMR Plus.

[8] Shoemaker M.E., Salmon O.F., Smith C.M., Duarte-Gardea M.O., Cramer J.T. (2022). Influences of vitamin D and iron status on skeletal muscle health: A narrative review. Nutrients.

[9] Holick M.F. (2007). Medical progress: Vitamin D deficiency. N. Engl. J. Med..

[10] Wacker M., Holick M.F. (2013). Vitamin D—Effects on skeletal and extraskeletal health and the need for supplementation. Nutrients.

[11] Zittermann A. (2003). Vitamin D in preventive medicine: Are we ignoring the evidence?. Br. J. Nutr..

[12] Cashman K.D., Dowling K.G., Škrabáková Z., Gonzalez-Gross M., Valtueña J., De Henauw S., Moreno L., Damsgaard C.T., Michaelsen K.F., Mølgaard C. (2016). Vitamin D deficiency in Europe: Pandemic?. Am. J. Clin. Nutr..

[13] Holick M.F., Binkley N.C., Bischoff-Ferrari H.A., Gordon C.M., Hanley D.A., Heaney R.P., Murad M.H., Weaver C.M. (2011). Evaluation, treatment, and prevention of vitamin D deficiency: An Endocrine Society clinical practice guideline. J. Clin. Endocrinol. Metab..

[14] Ross A.C., Manson J.E., Abrams S.A., Aloia J.F., Brannon P.M., Clinton S.K., Durazo-Arvizu R.A., Gallagher J.C., Gallo R.L., Jones G. (2011). The **2011** report on dietary reference intakes for calcium and vitamin D from the Institute of Medicine: What clinicians need to know. J. Clin. Endocrinol. Metab..

[15] Bikle D.D. (2014). Vitamin D metabolism, mechanism of action, and clinical applications. Chem. Biol..

[16] Christakos S., Dhawan P., Verstuyf A., Verlinden L., Carmeliet G. (2016). Vitamin D: Metabolism, molecular mechanism of action, and pleiotropic effects. Physiol. Rev..

[17] Bikle D.D. (2016). Extraskeletal actions of vitamin D. Ann. N. Y. Acad. Sci..

[18] Wang Y., Zhu J., DeLuca H.F. (2012). Where is the vitamin D receptor?. Arch. Biochem. Biophys..

[19] Bouillon R., Marcocci C., Carmeliet G., Bikle D., White J.H., Dawson-Hughes B., Lips P., Munns C.F., Lazaretti-Castro M., Giustina A. (2019). Skeletal and extraskeletal actions of vitamin D: Current evidence and outstanding questions. Endocr. Rev..

[20] Pilz S., Zittermann A., Trummer C., Theiler-Schwetz V., Lerchbaum E., Keppel M.H., Grübler M.R., März W., Pandis M. (2019). Vitamin D testing and treatment: A narrative review of current evidence. Endocr. Connect..

[21] Zmijewski M.A., Carlberg C. (2020). Vitamin D receptor(s): In the nucleus but also at membranes?. Exp. Dermatol..

[22] Costa E.M., Blau H.M., Feldman D. (1986). 1,25-dihydroxyvitamin D_3_ receptors and hormonal responses in cloned human skeletal muscle cells. Endocrinology.

[23] Bischoff H.A., Borchers M., Gudat F., Duermueller U., Theiler R., Stähelin H.B., Dick W. (2001). In situ detection of 1,25-dihydroxyvitamin D_3_ receptor in human skeletal muscle tissue. Histochem. J..

[24] DeLuca H.F. (2004). Overview of general physiologic features and functions of vitamin D. Am. J. Clin. Nutr..

[25] Wang Y., DeLuca H.F. (2011). Is the vitamin D receptor found in muscle?. Endocrinology.

[26] Girgis C.M. (2020). Vitamin D and skeletal muscle: Emerging roles in development, anabolism and repair. Calcif. Tissue Int..

[27] Hamilton B. (2010). Vitamin D and human skeletal muscle. Scand. J. Med. Sci. Sports.

[28] Pojednic R.M., Ceglia L. (2014). The emerging biomolecular role of vitamin D in skeletal muscle. Exerc. Sport Sci. Rev..

[29] de la Puente Yagüe M., Collado Yurrita L., Ciudad Cabañas M.J., Cuadrado Cenzual M.A. (2020). Role of vitamin D in athletes and their performance: Current concepts and new trends. Nutrients.

[30] Cannell J.J., Hollis B.W., Sorenson M.B., Taft T.N., Anderson J.J. (2009). Athletic performance and vitamin D. Med. Sci. Sports Exerc..

[31] Książek A., Zagrodna A., Słowińska-Lisowska M. (2019). Vitamin D, skeletal muscle function and athletic performance in athletes—A narrative review. Nutrients.

[32] Wiciński M., Adamkiewicz D., Adamkiewicz M., Śniegocki M., Podhorecka M., Szychta P., Malinowski B. (2019). Impact of vitamin D on physical efficiency and exercise performance—A review. Nutrients.

[33] Han Q., Li X., Tan Q., Shao J., Yi M. (2019). Effects of vitamin D3 supplementation on serum 25(OH)D concentration and strength in athletes: A systematic review and meta-analysis of randomized controlled trials. J. Int. Soc. Sports Nutr..

[34] Sist M., Zou L., Galloway S.D.R., Rodriguez-Sanchez N. (2023). Effects of vitamin D supplementation on maximal strength and power in athletes: A systematic review and meta-analysis of randomized controlled trials. Front. Nutr..

[35] Zhang L., Quan M., Cao Z.-B. (2019). Effect of vitamin D supplementation on upper and lower limb muscle strength and muscle power in athletes: A meta-analysis. PLoS ONE.

[36] Carswell A.T., Oliver S.J., Wentz L.M., Kashi D.S., Roberts R., Tang J.C., Izard R.M., Jackson S., Allan D., Rhodes L.E. (2018). Influence of vitamin D supplementation by sunlight or oral D3 on exercise performance. Med. Sci. Sports Exerc..

[37] Heileson J.L., McGowen J.M., Moris J.M., Chapman-Lopez T.J., Torres R., Funderburk L.K., Jeffrey S., Forsse J.S. (2022). Body composition, eicosapentaenoic acid, and vitamin D are associated with Army Combat Fitness Test Performance. J. Int. Soc. Sports Nutr..

[38] Laaksi A., Laaksi I., Pihlajamäki H., Vaara J.P., Luukkaala T., Kyröläinen H. (2023). Associations of serum 25(OH)D levels with physical performance and anabolic hormones in young men. Front. Physiol..

[39] Ööpik V., Timpmann S., Rips L., Olveti I., Kõiv K., Mooses M., Mölder M.H., Varblane M.A., Lille H.-R., Gapeyeva H. (2017). Anabolic adaptations occur in conscripts during basic military training despite high prevalence of vitamin D deficiency and decrease in iron status. Mil. Med..

[40] Andersen N.E., Karl J.P., Cable S.J., Williams K.W., Rood J.C., Young A.J., Lieberman H.R., McClung J.P. (2010). Vitamin D status in female military personnel during combat training. J. Int. Soc. Sports Nutr..

[41] Funderburk L.K., Daigle K., Arsenault J.E. (2015). Vitamin D status among overweight and obese soldiers. Mil. Med..

[42] Burden R.J., Morton K., Richards T., Whyte G.P., Pedlar C.R. (2015). Is iron treatment beneficial in, iron-deficient but non-anemic (IDNA) endurance athletes? A systematic review and meta-analysis. Br. J. Sports Med..

[43] Knapik J. (1989). The army physical fitness test (APFT): A review of the literature. Mil. Med..

[44] Kull M., Kallikorm R., Tamm A., Lember M. (2009). Seasonal variance of 25(OH)D in the general population of Estonia, a Northern European country. BMC Public Health.

[45] Koundourakis N.E., Androulakis N.E., Malliaraki N., Margioris A.N. (2014). Vitamin D and exercise performance in professional soccer players. PLoS ONE.

[46] Barringer N.D., Kotwal R.S., Lewis M.D., Funderburk L.K., Elliott T.R., Crouse S.F., Smith S.B., Greenwood M., Kreider R.B. (2016). Fatty acid blood levels, vitamin D status, physical performance, activity, and resiliency: A novel potential screening tool for depressed mood in active duty soldiers. Mil. Med..

[47] Aspray T.J., Bowring C., Fraser W., Gittoes N., Javaid M.K., Macdonald H., Patel S., Selby P., Tanna N., Francis R.M. (2014). National Osteoporosis Society vitamin D guideline summary. Age Ageing.

[48] Lee E.C., Fragala M.S., Kavouras S.A., Queen R.M., Pryor J.L., Casa D.J. (2017). Biomarkers in sports and exercise: Tracking health, performance, and recovery in athletes. J. Strength Cond. Res..

[49] Viru A., Viru M. (2001). Biochemical Monitoring of Sport Training.

[50] Chicharro J.L., López-Mojares L.M., Lucía A., Pérez M., Alvarez J., Labanda P., Calvo F., Vaquero A.F. (1998). Overtraining parameters in special military units. Aviat. Space Environ. Med..

[51] Salonen M., Huovinen J., Kyröläinen H., Piirainen J.M., Vaara J.P. (2019). Neuromuscular performance and hormonal profile during military training and subsequent recovery period. Mil. Med..

[52] Tait J.L., Drain J.R., Corrigan S.L., Drake J.M., Main L.C. (2022). Impact of military training stress on hormone response and recovery. PLoS ONE.

[53] Tanskanen M.M., Kyröläinen H., Uusitalo A.L., Huovinen J., Nissilä J., Kinnunen H., Atalay M., Häkkinen K. (2011). Serum sex hormone-binding globulin and cortisol concentrations are associated with overreaching during strenuous military training. J. Strength Cond. Res..

[54] Damas-Fuentes M., Boughanem H., Molina-Vega M., Tinahones F.J., Fernández-García J.C., Macías-González M. (2022). 25-hydroxyvitamin and testosterone levels association through body mass index: A cross-sectional study of young men with obesity. Front. Endocrinol..

[55] Nimptsch K., Platz E.A., Willett W.C., Giovannucci E. (2012). Association between plasma 25-OH vitamin D and testosterone levels in men. Clin. Endocrinol..

[56] D’Andrea S., Martorella A., Coccia F., Castellini C., Minaldi E., Totaro M., Parisi A., Francavilla F., Francavilla S., Barbonetti A. (2021). Relationship of vitamin D status with testosterone levels: A systematic review and meta-analysis. Endocrine.

[57] Wehr E., Pilz S., Boehm B.O., März W., Obermayer-Pietsch B. (2010). Association of vitamin D status with serum androgen levels in men. Clin. Endocrinol..

[58] Sircar S. (2008). Principles of Medical Physiology.

[59] Andrews N.C., Schmidt P.J. (2007). Iron homeostasis. Annu. Rev. Physiol..

[60] Umbreit J. (2005). Iron deficiency: A concise review. Am. J. Hematol..

[61] Beard J., Tobin B. (2000). Iron status and exercise. Am. J. Clin. Nutr..

[62] Haas J.D. (2006). The effects of iron deficiency on physical performance. Mineral Requirements for Military Personnel: Levels Needed for Cognitive and Physical Performance during Garrison Training.

[63] Moran D.S., Heled Y., Arbel Y., Israeli E., Finestone A.S., Evans R.K., Yanovich R. (2012). Dietary intake and stress fractures among elite male combat recruits. J. Int. Soc. Sports Nutr..

[64] O’Leary T.J., Jackson S., Izard R.M., Walsh N.P., Coombs C.V., Carswell A.T., Oliver S.J., Tang J.C.Y., Fraser W.D., Greeves J.P. (2024). Sex differences in iron status during military training: A prospective cohort study of longitudinal changes and associations with endurance performance and musculoskeletal outcomes. Br. J. Nutr..

[65] Yanovich R., Karl J.P., Yanovich E., Lutz L.J., Williams K.W., Cable S.J., Young A.J., Pasiakos S.M., McClung J.P. (2015). Effects of basic combat training on iron status in male and female soldiers: A comparative study. US Army Med. Dep. J..

[66] Martin N.M., Conlon C.A., Smeele R.J.M., Mugridge O.A.R., von Hurst P.R., McClung J.P., Beck K.L. (2019). Iron status and associations with physical performance during basic combat training in female New Zealand Army recruits. Br. J. Nutr..

[67] McClung J.P., Murray-Kolb L.E. (2013). Iron nutrition and premenopausal women: Effects of poor iron status on physical and neuropsychological performance. Annu. Rev. Nutr..

[68] Peeling P., Dawson B., Goodman C., Landers G., Trinder D. (2008). Athletic induced iron deficiency: New insights into the role of inflammation, cytokines and hormones. Eur. J. Appl. Physiol..

[69] Haas J.D., Brownlie T. (2001). 4th. Iron deficiency and reduced work capacity: A critical review of the research to determine a causal relationship. J. Nutr..

[70] Eichner E.R., Maughan R.J. (2001). Minerals: Iron. Nutrition in Sport.

[71] Masuda K., Okazaki K., Kuno S., Asano K., Shimojo H., Katsuta S. (2001). Endurance training under 2500-m hypoxia does not increase myoglobin content in human skeletal muscle. Eur. J. Appl. Physiol..

[72] Lundby C., Jacobs R.A. (2016). Adaptations of skeletal muscle mitochondria to exercise training. Exp. Physiol..

